# A Novel Sparse Representation Classification Method for Gas Identification Using Self-Adapted Temperature Modulated Gas Sensors

**DOI:** 10.3390/s19092173

**Published:** 2019-05-10

**Authors:** Aixiang He, Guangfen Wei, Jun Yu, Meihua Li, Zhongzhou Li, Zhenan Tang

**Affiliations:** 1 School of Information & Electronic Engineering, Shandong Technology and Business University, Yantai 264005, China; guangfen.wei@sdtbu.edu.cn (G.W.); limeihua@sdtbu.edu.cn (M.L.); 2 The Key Laboratory of Liaoning for Integrated Circuits Technology, Faculty of Electronic Information and Electrical Engineering, Dalian University of Technology, Dalian 116023, China; junyu@dlut.edu.cn (J.Y.); lizhongzhou127@163.com (Z.L.)

**Keywords:** electronic nose, gas identification, sparse representation classification (SRC), method of optimal directions (MOD), temperature modulation

## Abstract

A novel sparse representation classification method (SRC), namly SRC based on Method of Optimal Directions (SRC_MOD), is proposed for electronic nose system in this paper. By finding both a synthesis dictionary and a corresponding coefficient vector, the *i*-th class training samples are approximated as a linear combination of a few of the dictionary atoms. The optimal solutions of the synthesis dictionary and coefficient vector are found by MOD. Finally, testing samples are identified by evaluating which class causes the least reconstruction error. The proposed algorithm is evaluated on the analysis of hydrogen, methane, carbon monoxide, and benzene at self-adapted modulated operating temperature. Experimental results show that the proposed method is quite efficient and computationally inexpensive to obtain excellent identification for the target gases.

## 1. Introduction

Electronic noses are technical devices that contain a gas sensor array and pattern recognition system [1]. However, the pattern recognition of electronic noses, in many cases, is plagued with problems. It is quite usual to encounter drift, scattering due to concentration effects, highly correlated features, or non-Gaussian data distributions [2,3]. In addition, due to high calibration costs and complex experimental conditions, the number of training samples is limited. Hence, the performance of classifier is very important to electronic noses, as they can improve the robustness to the problems mentioned above.

At present, there are many classification methods for gas sensor data [4,5,6,7,8,9] such as deep learning and support vector machine (SVM). Since the concept of deep learning was put forward, it has attracted the attention of many scholars [10,11,12]. Peng et al. proposed a novel Deep Convolutional Neural Network (DCNN) tailored for gas classification [13]. Wei et al. also proposed a new improved LeNet-5 gas identification convolutional neural network structure for electronic noses [14].

Because support vector machine (SVM) has good generalization properties and robustness against the curse of dimensionality [15], SVM has been widely applied to gas identification [16,17,18]. Vergara et al. used Inhibitory Support Vector Machine (ISVM) to detect and identify odor under complex environmental conditions [17]. Sakumura et al. also used SVM to detect respiratory samples, and achieved high detection accuracy [18].

Other explored classification methods are artificial neural network [19], decision tree [20], and Bayesian Networks [21], etc. For example, Aleixandre used probabilistic neural network (PNN) and multilayer perceptrons (MLP) to discriminate four different pollutant gases [22]. Cho et al. used classification and regression tree to identify acetone, nitrobenzene, octane, and nitrotoluene, and obtained 94% classification accuracy [23].

Sparse representation classification (SRC) is proved to be robust to outliers, noise, and even incomplete measurements [24,25,26], and some scholars have successfully used SRC to solve the problem of gas classification, such as, Guo et al. [27] who used sparse representation-based classification to identify breath samples. However, SRC is time consuming, which limits its application.

The contribution of this paper is to propose a novel sparse representation classification method, namly SRC based on Method of Optimal Directions (SRC_MOD), to improve the classification performance of electronic nose. In order to improve the learning speed, the training set is divided into some subsets according to the label of samples, and the optimal solution of synthesis dictionary submatrix and coefficient submatrix are solved by MOD. Moreover, the testing phase is separated from the training phase. The structure of the article is as follows: Section 2 describes briefly introduces the experimental setup and data collection. Section 3 analyzes the proposed SRC_MOD method. Section 4 discusses comparison results with other classifiers. Section 5 presents the conclusions.

## 2. Experimental Set-Up and Data Collection

### 2.1. The Measurement Circuit

The selectivity and sensitivity of the metal oxide gas sensors can be improved by optimizing the operation temperature of the sensors [28,29]. Martinelli designed a self-adapted temperature modulation circuit and achieved high detection accuracy. This method implements the concept of self-adapted temperature modulation, and it is based on the evidence that the sensitivity to the gas of the sensor resistance depends on the operating temperature, and, conversely, the sensitivity to the temperature depends on the gas [28].

**Figure 1:**
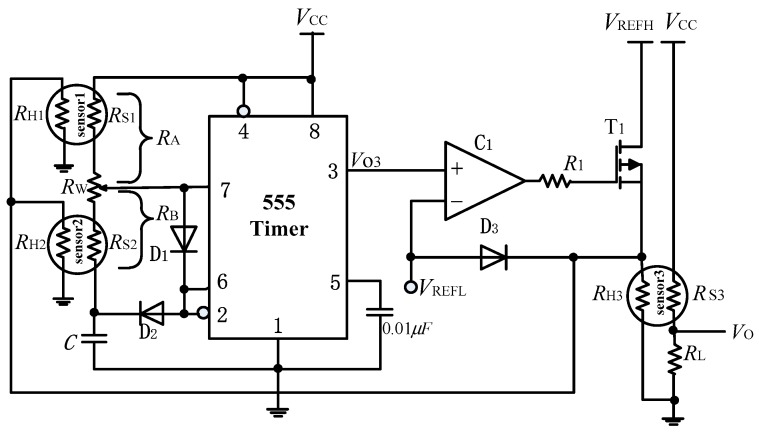
The measurement circuit. Sensor 1 is TGS2610, sensor 2 is TGS2610, and sensor 3 is TGS2620. *V_REFL_* and *V_REFH_* represent low and high reference voltage of heating voltage, respectively.

In this paper, we proposed an improved self-adapted temperature modulated measurement circuit to improve the performance of the electronic nose. The measurement circuit is shown in Figure 1. It mainly contains a multivibrator circuit, three gas sensors and a comparator *C*_1_. The resistances of sensor 1 and sensor 2 are part of the multivibrator circuit. *V_REFL_* and *V_REFH_* represent low and high reference voltage of heating voltage, respectively. In this paper, *V_REFL_* = 2 V and *V_REFH_* = 5 V.

We can see from Figure 1 that if the output voltage $V_{O3}$ of the 555 timer is low ($V_{O3}\approx 0$ V), $V_{O3}<V_{REFL}$, the output voltage of comparator *C*_1_ is low, and the transistor *T*_1_ will be cut off. When this happens, the heating voltage across sensor 3 or the three sensors? will be close to *V_REFL_*. On the other hand, if the output voltage $V_{O3}$ is high ($V_{O3}\approx V_{CC}$), $V_{O3}>V_{REFL}$, the output voltage of comparator $C_{1}$ is high, the transistor $T_{1}$ will be turned on. When this occurs, the heating voltage across sensor 3 or the three sensors? will be close to *V_REFH_*.

$V_{O3}$ is a square wave signal whose period depends on the capacitor *C* and the sensor resistances of sensor 1 and sensor 2. The charging time of capacitor *C* is given by:

(1) $$T_{1}=(R_{A}+R_{B})C\ln\frac{V_{CC}-V_{T-}}{V_{CC}-V_{T+}}$$

where, $V_{T+}=\frac{2}{3}V_{CC}$, $V_{T-}=\frac{1}{3}V_{CC}$.

And the discharge time of the capacitor *C* is given by:

(2) $$T_{2}=R_{B}C\ln\frac{0-V_{T+}}{0-V_{T-}}$$

hence, the period of square wave signal is:

(3) $$T=T_{1}+T_{2}=(R_{A}+2R_{B})C\ln2$$

and the duty cycle of the square wave signal *V*_O3_ is:

(4) $$q=\frac{R_{A}}{R_{A}+R_{B}}$$

The output voltage of the third sensor 3 is given by:

(5) $$V_{O}=\frac{R_{L}}{R_{S3}+R_{L}}V_{CC}$$

where, *R_S_*_3_ is the sensor resistor of the third TGS2620 sensor.

### 2.2. The Experimental Set-Up

**Figure 2:**
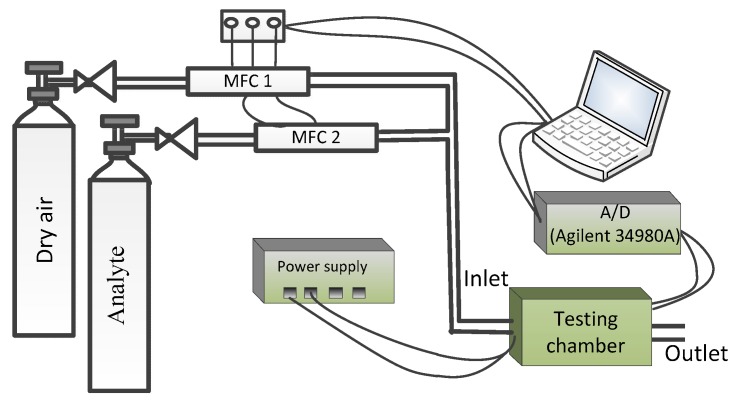
The experimental set-up.

Figure 2 shows the experimental set-up. The testing system uses two computer-controlled, digital mass flow controllers (MFCs). The testing gas at the desired concentration is conveyed to a 300 mL volume testing chamber by MFCs with highly reproducibility and higher accuracy. We keep the total flow constant for each test. In this paper, the total flow rate is set to 500 sccm.

The above-mentioned measurement circuit that contains three gas sensors (TGS2610, TGS2610, and TGS2620, Figaro, Inc. Japan) is placed into the testing chamber. In order to collect all experimental samples, a LabVIEW environment program running on a PC platform, and the sample frequency is set to 1 Hz.

The measurement procedure is as follows:

(1) Clean testing chamber with dry air for 50 s.
(2) The testing gas at the desired concentration is conveyed to the testing chamber by MFCs for 100 s.
(3) Clean the testing chamber with dry air for 100 s.

When the three sensors return to baseline steady-state response, repeat step 1 to 3 for the next test until all the experiments are completed.

### 2.3. Data Collection

Four chemical analytes with different concentrations are tested by the electronic nose system. As shown in Table 1, the tested gases are hydrogen, methane, carbon monoxide, and benzene. Each test is repeated 20 times, and finally 400 samples are collected.

**Figure 3:**
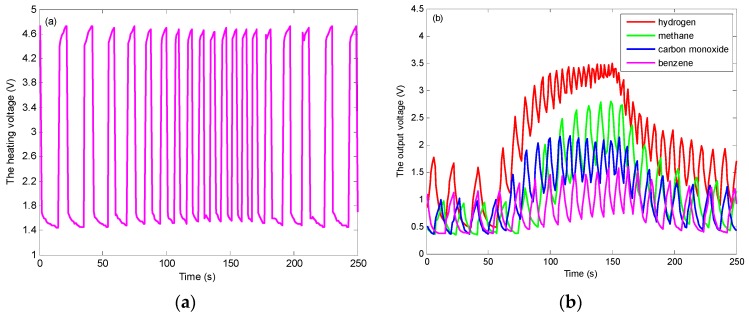
(**a**) The heating voltage of 30 ppm benzene; (**b**) The output voltages of 3000 ppm hydrogen, 3000 ppm methane, 300 ppm carbon monoxide, and 30 ppm benzene.

Figure 3 shows the heating voltage of 30 ppm benzene and the output voltages of four analytes. We can see from Figure 3a, the frequency of heating waveform in the middle is higher than that on both sides. The reason for this phenomenon is related to the change of resistances of sensor 1 and sensor 2. The reducing gas is injected into the testing chamber from the time of 51 to 150 s, which leads to the decrease of sensor resistances and the increase of waveform frequency. In this paper, setting *C* = 100 μF, the periods *T* of the heating voltage range from 2 to 25 s and the frequencies range from 40 to 500 mHz.

In this paper, 40 samples are choosed as a testing set, and the other samples for training and validation. In order to improve robustness of the algorithm, the classifiers use 10-fold cross-validation method. 360 samples are randomly divided into 10 subsets with equal size. A single subset is retained as the validation set, and the remaining nine subsets are used as training set. Hence, the number of validation samples is 36 and the number of training samples is 324. The program runs 10 times, with each of the 10 subsets used exactly once as the validation set. The prediction model with the highest recognition rate is used as the final model to identify the testing gas.

## 3. The Proposed SRC_MOD Algorithm for Gas Identification

Suppose $V_{O}\in R^{250\times 1}$ denotes a sensor signal that is a time-based variable and in total has 250 points. Firstly, the sensor siganl is removed additive noise or drift by $x=V_{O}-V_{ref}$, where, $V_{ref}$ is baseline steady-state output voltage in dry air. Then, the sensor sample x is normalized by x=(x−min(x))/(max(x)−min(x)), where, min() and max() denote the sample minimum and maximum value.

The normalized training sample dataset is represented by a matrix $X=[X_{1},X_{2},\cdots ,X_{n}]\in R^{250\times 324}$, where, $X_{i}$, i=1,2,⋯,n, is a submatrix of training sample corresponding to class *i* and each column is a sensor sample, *n* is total number of categories.

The *i*th class training samples $X_{i}$ is approximated as a linear combination of some few of the dictionary atoms. The approximation $X_{i}^{*}$ can be written as:

(6) $$X_{i}^{*}=D_{i}W_{i}\begin{array}{ccc} & s.t. & \min{\left\|W_{i}\right\|}_{0} \end{array}$$

where, ${\left\|\cdot \right\|}_{0}$ is $l_{0}$-norm, $W_{i}$ is the coefficients of the *i*th class training samples and most of the entries in $W_{i}$ are zero, $D_{i}$ is a synthesis dictionary corresponding to class *i*. Equation (6) describes each given sensor signal as the sparsest representation $W_{i}$ over the synthesis dictionary $D_{i}$, and aims to jointly find the proper representations and the dictionary. If a solution has been found such that every representation has fewer non-zero entries, a candidate feasible model has been found. In this paper, the synthesis dictionary *D_i_* is initialized as a random matrix with the size of 250 × 250.

Equation (6) can be formulated as an optimization problem with respect to $W_{i}$ and $D_{i}$. With γ, we may put it as:

(7) $$\left\{D_{i},W_{i}\right\}=\arg\underset{D_{i},W_{i}}{\min}({\left\|X_{i}-D_{i}W_{i}\right\|}_{2}^{2}+\gamma {\left\|W_{i}\right\|}_{0})$$

As γ increases, the solution is getting more dense. Solutions of Equation (7) can be found by the Method of Optimal Directions (MOD). MOD is a dictionary learning algorithm [30]. It’s aim is to find both a dictionary *D_i_* and a corresponding coefficient matrix *W_i_* such that the representation error $R=X_{i}-D_{i}W_{i}$ is minimized and *W_i_* fulfill some sparseness criterion. The procedures of obtaining the optimal solution of *D_i_* and *W_i_* are summarized in Algorithm 1.


**Algorithm 1. Obtain the optimal solution of**
$D_{i}$
**and**
$W_{i}$
Input: The *i*-th class training samples $X_{i}$, maximum error ε, *k* = 1.        1. Initialize dictionary $D_{i}^{\left(0\right)}$ and $W_{i}^{\left(0\right)}$ with two random matrices.        2. *while* (error>ε)          update $D_{i}^{\left(k+1\right)}=X_{i}{\left(W_{i}^{\left(k\right)}\right)}^{T}{\left(W_{i}^{\left(k\right)}{\left(W_{i}^{\left(k\right)}\right)}^{T}\right)}^{-1}$;          update $W_{i}^{\left(k+1\right)}=\arg\underset{W_{i}}{\min}({\left\|X_{i}-D_{i}^{\left(k\right)}W_{i}^{\left(k\right)}\right\|}_{2}^{2})$ by pursuit algorithm [31];          $error={\left\|X_{i}-D_{i}^{\left(k\right)}W_{i}^{\left(k\right)}\right\|}_{F}^{2}$;          k←k+1;          *end while* Output: The *i*-th class synthesis dictionary $D_{i}$ and coefficient matrix $W_{i}$.

For ∀i, the training samples X can be projected onto a coding coefficient space via $P_{i}X$, where $P_{i}$ is an analysis dictionary corresponding to class *i*. The coding coefficient matrix *W_i_* is given by:

(8) $$W_{i}=P_{i}X_{i}$$

where, $P_{i}$ is a full-rank matrix. If most of large coefficients generated by $P_{i}X$ are concentrated in $W_{i}$, while the coding coefficients of the other class training samples over $P_{i}$ is as small as possible, the discrimination power of $P_{i}$ can be promoted. Hence, we may improve the discrimination power of $P_{i}$ by min ${\left\|P_{i}{\bar{X}}_{i}\right\|}_{F}^{2}$, where, ${\bar{X}}_{i}$ is the complementary data matrix of $X_{i}$ in the whole training set X and ${\left\|\cdot \right\|}_{F}$ is the Hilbert-Schmidt norm or the Frobenius norm.

We evaluate the error using a Frobenius norm. The *i*-th class analysis dictionary *P_i_* can be approximated by

(9) $$P_{i}=\arg\underset{P_{i}}{\min(}{\left\|W_{i}-P_{i}X_{i}\right\|}_{F}^{2}+\alpha {\left\|P_{i}{\bar{X}}_{i}\right\|}_{F}^{2}+\beta {\left\|P_{i}\right\|}_{F}^{2})$$

where, the first term minimizes the error of the *i*th class coding coefficients, the second term is used to improve the recognition performance of the analysis dictionary $P_{i}$, and α is a scalar constant. The third term is to avoid a high risk of overfitting to training samples, and β is a regularization parameter.

We then solve for $P_{i}$ by least-squares. Differentiating Formula (9) with respect to $P_{i}$, such a differentiation results in:

(10) $$\begin{array}{c} \frac{\partial }{\partial P_{i}}({\left\|W_{i}-P_{i}X_{i}\right\|}_{F}^{2}+\alpha {\left\|P_{i}{\bar{X}}_{i}\right\|}_{F}^{2}+\beta {\left\|P_{i}\right\|}_{F}^{2})\\ =2(P_{i}X_{i}-W_{i})X_{i}^{T}+2\alpha P_{i}{\bar{X}}_{i}{\bar{X}}_{i}^{T}+2\beta P_{i} \end{array}$$

Setting Equation (10) equal to zero gives the optimum $P_{i}$ as:

(11) $$P_{i}=(W_{i}X_{i}^{T}){\left(X_{i}X_{i}^{T}+\alpha {\bar{X}}_{i}{\bar{X}}_{i}^{T}+\beta I\right)}^{-1}$$

Since $W_{i}=P_{i}X_{i}$, Equation (6) can also be given by

(12) $$X_{i}^{*}=D_{i}P_{i}X_{i}$$

Define $\Phi_{i}=D_{i}P_{i}$ as a projection matrix, and the approximation $X_{i}^{*}$ is then rewritten as

(13) $$X_{i}^{*}=\Phi_{i}X_{i}$$

Using Equation (13), an arbitrary testing sample $x_{test}$ can be reconstructed as:

(14) $$x_{i}^{*}=\Phi_{i}x_{test}$$

We obtain *n* approximations $x_{1}^{*},x_{2}^{*},\cdots ,x_{n}^{*}$, and calculate the residual between $x_{test}$ and $x_{i}^{*}$ by

(15) $$Label(x_{test})=\arg\underset{i}{\min}{\left\|x_{i}^{*}-x_{test}\right\|}_{2}^{2}$$

where, ${\left\|\cdot \right\|}_{2}$ is *l*_2_-norm. The label corresponding to the minimum residual is the class of the testing sample.

**Figure 4:**
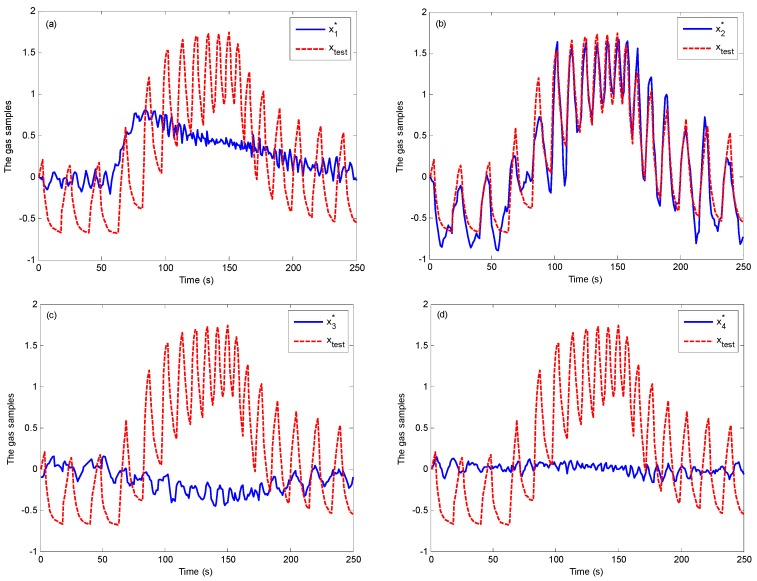
The original testing sample and four reconstrcted samples for 1000 ppm methane (**a**) The original testing sample $x_{test}$ and the 1th class reconstrcted sample $x_{1}^{*}$; (**b**) The original testing sample $x_{test}$ and the 2th class reconstrcted sample $x_{2}^{*}$; (**c**) The original testing sample $x_{test}$ and the 3th class reconstrcted sample $x_{3}^{*}$; (**d**) The original testing sample $x_{test}$ and the 4th class reconstrcted sample $x_{4}^{*}$.

**Figure 5:**
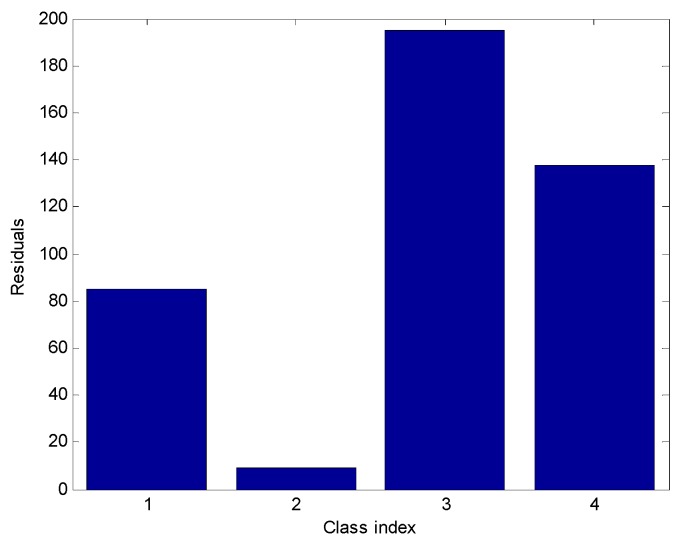
Residuals between the original sample and reconstructed samples.

Figure 4 shows the original testing sample $x_{test}$ and four reconstrcted samples $x_{i}^{*}$, (i=1,2,3,4). $x_{i}^{*}$ is obtained by Formula (14). From Figure 4, we can see that $x_{2}^{*}$ is closest to the original testing sample $x_{test}$ The residuals between $x_{test}$ and $x_{i}^{*}$, (i=1,2,3,4) are represented in Figure 5. From Figure 5, we can see that the second residual is the smallest. Hence, the testing sample $x_{test}$ is the 2nd class, namely methane.

The proposed SRC_MOD algorithm is summarized in Algorithm 2.


**Algorithm 2. The proposed SRC_MOD algorithm**
Input: The training samples for *n* classes, testing samples.      1. *for i* = *1*:*n*              obtain $D_{i}$ and $W_{i}$ by Algorithm 1;              obtain $P_{i}$ by Equation (11);          *end for*      2. $\Phi_{i}=D_{i}P_{i}$      3. Reconstructing the testing sample by Equation (14)      4. To identify the testing sample by Equation (15) Output: The label of the testing sample.

## 4. Comparisons with Other Classifiers

In order to evaluate the performance of the proposed algorithm, we compare it with other algorithms, such as SRC (used in [27]), the dictionary learning (DL) classifier (proposed in [32]), deep learning (used in [14]) and BP artificial neural network. All experiments in this paper run on a dual-core processor with a CPU main frequency of 2.4 GHz. Python is applied for deep learning and MATLAB for the other algorithms. Accuracy and processing time are the average values of all testing samples.

For SRC, the algorithm is the same as that in [27]. At first, the testing sample is approximated as a linear combination of all training samples

(16) $$x_{test}=WX_{train}$$

where, W is a sparse coefficient vector, $X_{train}$ is a matrix of all training samples.

The sparsest solution of Equation (16) is defined as the following an *l*_1_-minimization problem:

(17) $$W=\arg\underset{{}_{W}}{\min}({\left\|WX_{train}-x_{test}\right\|}^{2}+\lambda {\left\|W\right\|}_{1})$$

where, ${\left\|\cdot \right\|}_{1}$ is *l*_1_-norm, λ is the regularization parameter. The solution to the *l*_1_-minimization problem can also be obtained by using the MATLAB package provided by Reference [33]. Keep the *i*th class coefficients and set the other coefficients equal to zero. We have

(18) $$W_{i}^{*}=\{0,0,W_{i},\cdots ,0\}$$

Reconstruct the testing sample by $x_{i}^{*}=\;W_{i}^{*}X_{train}$, (i=1,2,3,4) and use Equation (15) to obtain the class of the testing sample.

For DL classifier, the *i*-th class analysis dictionary, coding coefficients, and synthesis dictionary are trained together to generate the prediction model. It is a simple and effective dictionary learning algorithm. This is our previous work and more details of the algorithm are shown in [32].

**Figure 6:**
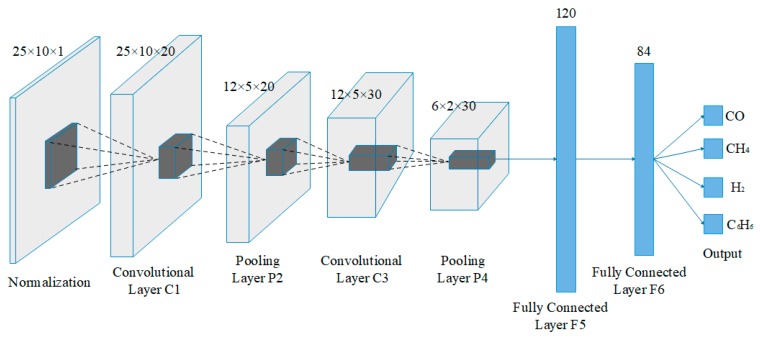
The LeNet-5 structure.

For deep learning, as shown in Figure 6, a LeNet-5 convolutional neural network structure is built. C1 and C3 are convolutional layers with kernel size of 3 × 3 and 2 × 2, respectively. C1 computes 20 filters over its input. The first convolutional layer C1 takes a matrix with size of 25 × 10 × 1 and outputs a matrix with size of 25 × 10 × 20. Pooling layer P2 and P4 are all done with 2 × 2 windows. Max pooling consists of extracting windows from the input features and outputting the max value of each channel. Before the first max-pooling layer P2, the feature map is 25 × 10, but the pooling operation halves it to 12 × 5. The numbers of fully connected layer F5 and F6 are 120 and 84, respectively. Finally, the label of gas sample is obtained. This is also our previous work and more details are shown in [14].

For the BP artificial neural network, the structure of the BP network is set to 250-501-4. The transfer function ‘tansig’ is applied for hidden layer, and ‘purelin’ for output layer.

The experimental results of the proposed SRC_MOD method are shown in Table 2. From Table 2, we can see that the average accuracy of SRC_MOD is about 98.44%, the average training time is nearly 0.2061 s and the average testing time is nearly 3.1 ms. SRC_MOD classifier obtains high accuracy in short testing time. Hence, the performance of the SRC_MOD classifier is perfect.

The experimental results of other classifiers are also shown in Table 2. The average accuracy of SRC is about 98.52% and the average testing time is nearly 1987.9 ms. The testing time is 641 times longer than that of SRC_MOD. The main reason is that training and testing of SRC are conducted simultaneously, solving *l*_1_-minimization problem is time consuming, repeatedly training for each test leads to longer processing time.

The average accuracy of DL classifier is about 96.88% and the testing time is nearly 6.5 ms. We find that SRC_MOD method is superior to the DL classifier in both recognition accuracy and testing time. Since alternating direction method of multipliers(ADMM) is more complex than MOD when obtaining the optimal solution of *D_i_*.

The average accuracy of deep learning is about 91.87%, the average training time is nearly 12.84 s and testing time is nearly 23.5 ms. The performance of deep learning significantly worse than SRC_MOD method. The main reason is that deep learning is more suitable for large training sets. In this experiment, the size of training samples is too small to show its advantage. For BP artificial neural network, the performance significantly worse than the other classifiers.

In a word, comparison results show that the proposed SRC_MOD method is quite efficient and computationally inexpensive to obtain excellent identification for the target gases.

## 5. Conclusions

This paper presents a SRC_MOD gas recognition algorithm. First the *i*-th class training samples are approximated as a linear combination of the synthesis dictionary atoms. Next, MOD is applied to solve the optimal solution of the synthesis dictionary and coefficient vector. Finally, we obtain the analysis dictionary and establish the prediction model. Compared with other classical classifiers (such as SRC, dictionary learning classifier, deep learning and BP artificial neural network), the experimental results show that SRC_MOD has better performance, not only in recognition rate but also in testing speed.

## Figures and Tables

**Table 1:** Dataset in detail.

Label	Analyte	Concentration (ppm)	Number
1	hydrogen	1000, 2000, 3000, 4000, 5000	100
2	methane	1000, 2000, 3000, 4000, 5000	100
3	carbon monoxide	100, 200, 300, 400, 500	100
4	benzene	10, 15, 20, 25, 30	100
Total			400

**Table 2:** Performance comparison with other algorithms.

Algorithm	Accuracy (%)	Training Time (s)	Testing Time (ms)
SRC_MOD	98.44	0.2061	3.1
SRC	98.52	no need	1987.9
DL	96.88	0.8061	6.5
Deep learning	91.87	12.84	23.5
BP	84.51	6.399	12.7
